# Active Learning Plus Deep Learning Can Establish Cost-Effective and Robust Model for Multichannel Image: A Case on Hyperspectral Image Classification

**DOI:** 10.3390/s20174975

**Published:** 2020-09-02

**Authors:** Fangyu Shi, Zhaodi Wang, Menghan Hu, Guangtao Zhai

**Affiliations:** 1 Institute of Image Communication and Information Processing, Shanghai Jiao Tong University, Shanghai 200240, China; fangyu.shi@sjtu.edu.cn (F.S.); wangzhaodi@sjtu.edu.cn (Z.W.); mhhu@ce.ecnu.edu.cn (M.H.); 2 Shanghai Key Laboratory of Multidimensional Information Processing, East China Normal University, Shanghai 200241, China; 3 Key Laboratory of Artificial Intelligence, Ministry of Education, Shanghai 200240, China

**Keywords:** deep learning, active learning, multichannel image, cost-effective model, hypersepctral image

## Abstract

Relying on large scale labeled datasets, deep learning has achieved good performance in image classification tasks. In agricultural and biological engineering, image annotation is time-consuming and expensive. It also requires annotators to have technical skills in specific areas. Obtaining the ground truth is difficult because natural images are expensive. In addition, images in these areas are usually stored as multichannel images, such as computed tomography (CT) images, magnetic resonance images (MRI), and hyperspectral images (HSI). In this paper, we present a framework using active learning and deep learning for multichannel image classification. We use three active learning algorithms, including least confidence, margin sampling, and entropy, as the selection criteria. Based on this framework, we further introduce an “image pool” to make full advantage of images generated by data augmentation. To prove the availability of the proposed framework, we present a case study on agricultural hyperspectral image classification. The results show that the proposed framework achieves better performance compared with the deep learning model. Manual annotation of all the training sets achieves an encouraging accuracy. In comparison, using active learning algorithm of entropy and image pool achieves a similar accuracy with only part of the whole training set manually annotated. In practical application, the proposed framework can remarkably reduce labeling effort during the model development and upadting processes, and can be applied to multichannel image classification in agricultural and biological engineering.

## 1. Introduction

Deep convolutional neural networks (CNNs) have achieved outstanding performance in image classification tasks, not only due to sufficient computing power and well-trained models, but also thanks to large scale annotated datasets, such as ImageNet [1], Open Image [2] and PASCAL VOC [3]. For natural images, manual annotation work, which is tedious and time-consuming, can be accomplished by people with limited training. However, in agricultural and biological engineering, obtaining the ground truth is time-consuming and expensive. It also requires annotators to possess technical skills in specific areas.

Active learning can achieve better performance with fewer annotated training data since it chooses more informative data to learn [4]. The active learner poses queries according to specific criteria, then the selected unlabeled data is annotated by human annotators. When the unlabeled data are abundant and it is costly to obtain the labels, active learning can possibly build a cost-effective model to significantly reduce the annotation cost.

Thus, we now aim to establish a framework to remarkably reduce the annotation cost without lowering the classification performance, using active learning and deep learning for multichannel image classification.

### 1.1. Related Work

#### 1.1.1. Active Learning

The main idea of active learning is to select the most informative unlabeled samples and avoid unnecessary manual annotation [4]. Therefore, the essential of active learning is the selection strategy, namely choosing samples to be manually annotated.

Active learning methods based on informativeness select samples with a high degree of uncertainty. Based on the number of involved models, this method can also be further subdivided into uncertainty sampling [5] (e.g., least confident [5,6], margin sampling [7] and entropy-based [8]) and query-by-committee (QBC) [9]. In uncertainty sampling, the learner queries the instances about which it is least certain how to label. In QBC, the learner randomly selects several hypotheses from the version space to form a committee, whose composition can be optimized by the classifier integration algorithm such as Bagging, AdaBoost, etc. [10]. The committee then chooses the most divergent examples for manual annotation.

In existing studies, active learning is usually combined with a special classifier, such as support vector machine (SVM) [11], logistic regression [12], and Gaussian process regression [13].

#### 1.1.2. Apply Deep Learning on Multichannel Image

Some researchers had attempted to apply deep learning to hyperspectral images. Noor et al. proposed image enhancement algorithms that can be used to improve the interpretability of data into clinically relevant information to facilitate diagnostics [14]. Liu et al. used CNN for analyzing hyperspectral data, indicating that the deep learning framework can give excellent performance for detection of defect regions on surface-defective cucumbers [15]. Jeon and Hu et al. employed deep CNN to classify hyperspectral remote sensing images in spectral domain [16]. Li et al. proposed a novel pixel-pair method to significantly increase the training data [17].

#### 1.1.3. Using Active Learning and Deep Learning in Combination

A few scholars have proposed to combine active learning and deep learning. Wang and Shang were the first to apply active learning in deep learning, using one of three metrics for data selection: least confidence, margin sampling and entropy [18]. Wang et al. proposed a novel active learning framework called CEAL (cost-effective active learning), building a competitive classifier with optimal feature representation with a limited amount of labeled training instances in an incremental learning manner [19]. Sener et al. defined the problem of active learning as core-set selection and presented a theoretical result characterizing the performance of any selected subset using the geometry of the datapoints [20]. Zhou et al. proposed a semi-supervised learning algorithm called active deep network (ADN) [21].

Based on the combination of active learning and deep learning, some researchers aim to solve different kinds of image tasks. In face identification, Lin et al. combined active learning and self-paced learning, automatically annotating new instances and incorporating them into training sets under weak expert recertification [22]. In biomedical image classification, Zhou et al. proposed a novel method called AIFT (active, incremental fine-tuning), integrating active learning and transfer learning into a single framework which reduces annotation cost [23]. In ground objects identification using hyperspectral remote sensing, Liu et al. utilized active learning and deep belief network (DBN), achieving a higher accuracy by actively selecting ferwer training samples [24]. Al Rahhal et al. proposed a novel approach based on deep learning for active classification of electrocardiogram (ECG) signals to deal with insufficient labeled data in natural language processing community [25]. To the best of our knowledge, there is no publication on the tandem use of active learning and deep learning for multichannel images.

### 1.2. Contribution of this Work

In this work, in order to solve the problem of expensive annotated datasets in agricultural and biological engineering, we present a framework for multichannel images, using active learning algorithm and deep learning framework with an “image pool”. In addition, when data augmentation is implemented, we deal with the situation where multiple images share labels, further reducing the annotation cost remarkably. We present a case study using blueberry dataset based on hyperspectral transmittance images, proving the availability of the proposed framework.

## 2. Method

### 2.1. Active Learning Selection Criteria

By introducing active learning into this study, we attempt to select the most informative instances in the training process, rather than randomly or exhaustively acquiring all the training instances. To select informative images as the training set, we introduce three active learning criteria, i.e., least confidence, margin sampling, and entropy.

In the kth training iteration, we define the CNN output probability that image $y_{i}$ belongs to jth category as $p(y_{i}=j|x_{i};W^{\left(k\right)})$. The confidence *C* of image $y_{i}$ using three selection criteria respectively is described as follows.

The least confidence algorithm evaluates the probability of the most likely category for a image. The lower the confidence is, the more uncertain that model classifies this image. This criterion only considers the most probable label, discarding the information of the remaining samples.

(1) $$C_{i}^{\left(k\right)}=\max_{j}p\left(y_{i}=j|x_{i};W^{\left(k\right)}\right)$$

The margin sampling algorithm ranks the confidence by the difference of the top two predicted categories. The smaller the difference is, the more difficult for the model to distinguish between the two categories. Margin sampling improves least confidence by incorporating the posterior of the top two likely label.

(2) $$C_{i}^{\left(k\right)}=p\left(y_{i}=j_{1}|x_{i};W^{\left(k\right)}\right)-p\left(y_{i}=j_{2}|x_{i};W^{\left(k\right)}\right)$$

where $j_{1}$ and $j_{2}$ are the top two most likely predicted categories.

The entropy-based algorithm of ranks the confidence by information entropy, taking all the classes into consideration. Entropy is a measure of information theory, which represents the amount of information required for encoding a distribution. Therefore, it is generally considered as a measure of uncertainty.

(3) $$C_{i}^{\left(k\right)}=\sum_{j=1}^{m}p\left(y_{i}=j|x_{i};W^{\left(k\right)}\right)\log p\left(y_{i}=j|x_{i};W^{\left(k\right)}\right)$$

For binary classification, the above three algorithms are equivalent, querying the instance with a class posterior closest to 0.5.

In each iteration, all the unlabeled images are sorted according to the confidence level. We believe that the current classifier has not yet well learned the characteristics of images with low confidence, thus they are more informative for the classifier and require manual annotation. Images with high certainty are learned well by the model, so they are pseudo annotated according to the output probability of the fine-tuned CNN of the last iteration.

### 2.2. Principle of Proposed Framework

We define $p^{\left(i\right)}$ as the percentage of pseudo-labeled training images in iteration *k*. The number of pseudo-labeled image $N_{pseudo}$ in iteration *k* is:

(4) $$N_{pseudo}^{\left(k\right)}=\left\lfloor N_{train}\times p^{\left(k\right)}\right\rfloor$$

where $N_{train}$ is the number of images in training set.

CNN will increasingly learn more about the input data with the training process. Therefore, it is reasonable to use the ascending amount of pseudo-labeled data as the model is trained. We define $p^{\left(k+1\right)}$ as:

(5) $$p^{\left(k+1\right)}=p^{\left(k\right)}+\delta \cdot k$$

where δ is the stride length of *p*.

The algorithm is illustrated in Algorithm 1.
**Algorithm 1** Active deep learning for multichannel images**Input:**$X_{train}$: Training set.$X_{test}$: Testing set.*K*: Number of manually annotated samples in each epoch.$p^{\left(0\right)}$: Initial percentage of pseudo labeled images from the unlabeled images.δ: Stride length of *p*.$X_{M}$: Manually labeled image set.$X_{P}$: Pseudo labeled image set.$X_{U}$: Unlabeled image set.**Output:**W: Fine-tuned CNN model.$X_{M}$: Manually labeled images.**Initialize:**Randomly select *K* images from $X_{train}$, and add them to $X_{M}^{\left(0\right)}$.$X_{U}^{\left(0\right)}\leftarrow X_{train}-X_{M}^{\left(0\right)}$, $X_{P}^{\left(0\right)}\leftarrow ⌀$.Fine-tune the CNN model and get $W^{\left(0\right)}$.1:**repeat**2: Sort high to low the certainty of images in $X_{U}^{\left(k\right)}$ according to *C* calculated by Equation (1), Equation (2) or Equation (3).3: Add top $N_{pseudo}^{\left(k\right)}$ images into $X_{P}^{\left(k\right)}$ for pseudo labeling based on Equation (4).4: Add last *K* images into $X_{M}^{\left(k\right)}$ for manually annotation.5: Use $X_{M}^{\left(k\right)}\cup X_{P}^{\left(k\right)}$ as the training set in this iteration.6: Fine-tune the CNN model and get $W^{\left(k\right)}$.7: Update *p* according to Equation (5).8: $X_{U}^{\left(k+1\right)}\leftarrow X_{U}^{\left(k\right)}\cup X_{P}^{\left(k\right)}$
9: $X_{P}^{\left(k+1\right)}\leftarrow ⌀$
10:**until** Meet specific criteria or run out the budget.11:**return**W and $X_{M}$.

**Figure 1:**
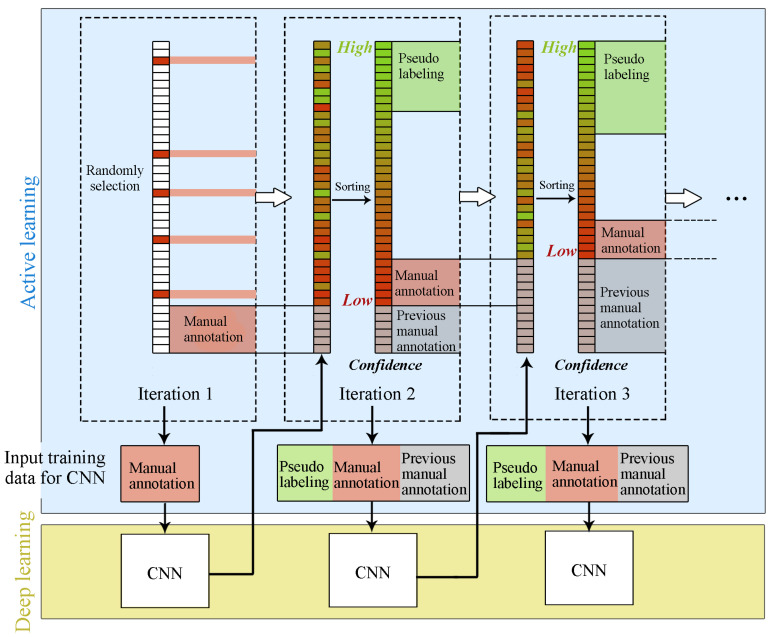
The application of active learning in the proposed framework.

Figure 1 presents the flow diagram of the framework in Algorithm 1.

### 2.3. Taking Full Advantage of Images Generated by Data Augmentation

Data augmentation is frequently used to boost the performance of deep CNN when the amount of original data is insufficient. Data augmentation creates training images using different ways of processing or combining multiple processing methods, such as random rotation, shifts, shear and flips, etc.

With one image, several associated images can be generated with data augmentation, and all of these images belong to a same blueberry sample. Therefore, when an image is manually annotated, the associated images obtain their labels in the meantime. It would be unwise if we ignore such characteristics.

**Figure 2:**
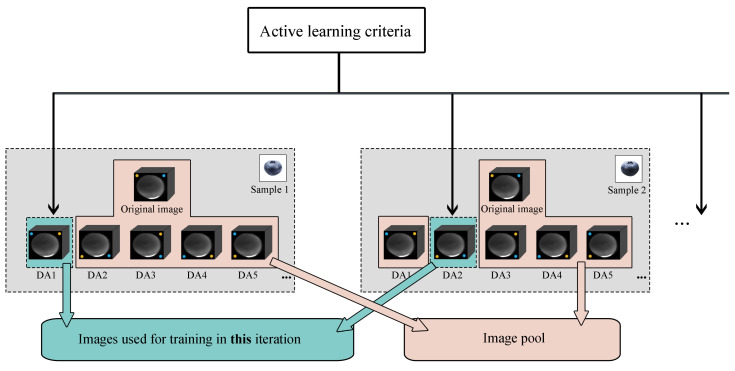
The principle of the image pool. “DA1”, “DA2”, etc. in the figure represent images generated by different data augment methods. Images in the image pool are used for the training in next iterations.

We define an “image pool” to store the associated images for the training of next iteration. After *K* most informative training images are selected by the active learning criteria, we add the associated images into the image pool Pool. When the number of images in Pool reaches *K*, no new images are manually annotated in the next iteration. Instead, *K* random images from Pool are used as the manually labeled images for the training process of next iteration. Figure 2 presents the principle of the image pool.

The algorithm using an image pool is illustrated in Algorithm 2. The result shows that this improvement dramatically reduces the amount of annotated images while guarantee the prediction accuracy comparing with Algorithm 1.
**Algorithm 2** Active deep learning for multichannel images using image pool**Input:**$X_{train}$: Training set.$X_{test}$: Testing set.*K*: Number of manually annotated samples in each epoch.$p^{\left(0\right)}$: Initial percentage of pseudo labeled images from the unlabeled images.δ: Stride length of *p*.$X_{M}$: Manually labeled image set.$X_{P}$: Pseudo labeled image set.$X_{U}$: Unlabeled image set.Pool: Image pool.**Output:**W: Fine-tuned CNN model.$X_{M}$: Manually labeled images.**Initialize:**Randomly select *K* images from $X_{train}$, and add them to $X_{M}^{\left(0\right)}$.$X_{U}^{\left(0\right)}\leftarrow X_{train}-X_{M}^{\left(0\right)}$, $X_{P}^{\left(0\right)}\leftarrow ⌀$, $Pool^{\left(0\right)}\leftarrow ⌀$.Fine-tune the CNN model and get $W^{\left(0\right)}$.1:**repeat**2: Sort high to low the certainty of images in $X_{U}^{\left(k\right)}$ according to *C* calculated by Equation (1), Equation (2) or Equation (3).3: Add top $N_{pseudo}^{\left(k\right)}$ images into $X_{P}^{\left(k\right)}$ for pseudo labeling based on Equation (4).4: **if** Card($Pool^{\left(k\right)}$)<K **then**5:  Add last *K* images into $X_{M}^{\left(k\right)}$ for manually annotation.6:  Add the associated images of $X_{M}^{\left(k\right)}$ into $Pool^{\left(k\right)}$.7: **else**8:  Randomly move *K* images from $Pool^{\left(k\right)}$ to $X_{M}^{\left(k\right)}$.9: **end if**10: Use $X_{M}^{\left(k\right)}\cup X_{P}^{\left(k\right)}$ as the training set in this iteration.11: Fine-tune the CNN model and get $W^{\left(k\right)}$.12: Update *p* according to Equation (5).13: $X_{U}^{\left(k+1\right)}\leftarrow X_{U}^{\left(k\right)}\cup X_{P}^{\left(k\right)}$
14: $X_{P}^{\left(k+1\right)}\leftarrow ⌀$
15:**until** Meet specific criteria or run out the budget.16:**return**W and $X_{M}$.

## 3. Result and Disscussion

### 3.1. Feasibility and Advantage of Using Deep Learning for Hyperspectral Image

Since the blueberry skin is composed of deep dark pigments, the pulp and other tissues under the skin are invisible to the naked eye. Hence, it has been considered a challenging task to utilize the RGB imaging technique and human eye detection method to accurately screen out berries with mechanical damage underneath the skin. Moreover, for manual inspection by human eye, the procedure is time-consuming and error-prone.

Zhang et al. validated the feasibility of hyperspectral transmittance imaging mode for quantifying blueberry bruises [26]. Hu et al. compared the performances of hyperspectral reflectance, transmittance and interactance imaging modes for detection of sightless blueberry damage demonstrating that the hyperspectral transmittance imaging mode was identified to be more sensitive to sightless blueberry damage than reflectance and interactance modes [27].

In the previous study, we have introduced deep learning techniques into the classification tasks of agricultural engineering based on the hyperspectral transmittance images, achieving better performance than traditional machine learning methods and proving the feasibility of using CNN to solve multichannel image classification task [28].

### 3.2. Dataset Description

We collected blueberries from Frutera S.A., Chile. To guarantee the model robustness, only blueberries with little visible physical damage and sound surface were used for analysis [29]. Therefore, a total of 575 blueberries including 304 sound samples and 253 damaged samples were applied for the following experiments.

**Figure 3:**
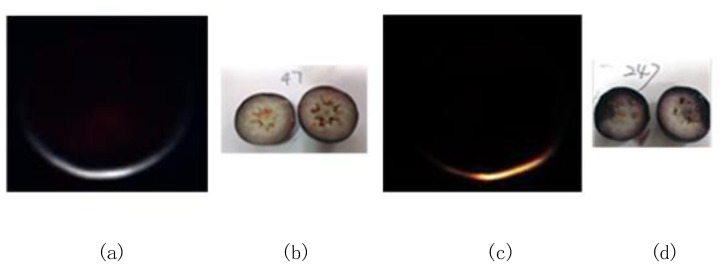
Hyperspectral transmittance images of the sound (**a**) and damaged (**c**) samples and their corresponding ground truth information ((**b**,**d**) for the sound and damaged samples, respectively).

All blueberries were cut through equator (Figure 3b,d) to obtain the ground truth, since the internal mechanical damage of blueberry was invisible. It is difficult to distinguish between the sound and the damaged with the naked eye when blueberries have not yet been cut. According to the damage degrees, the damaged areas more than 25% of cut surface were classified as the damaged category.

**Figure 4:**
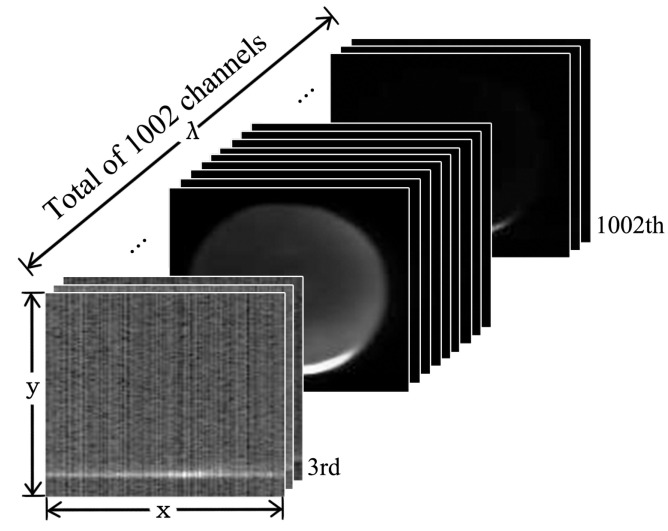
Data structure of the original hyperspectral image cube, where *x*, *y* and λ denote spatial *x*-axis, spatial *y*-axis, and spectral λ-axis, respectively.

Figure 4 shows the data structure of hyperspectral transmittance image cube. The width and height of images vary from 100 to 130 pixels. Each image cube contains 1002 spectral channels, whose wavelengths vary from 328.82 nm to 1113.54 nm, with incensement of 0.72 nm to 0.81 nm.

In this study, we randomly select 80% of the samples as training set, while the remaining part are the testing set.

### 3.3. Data Pre-Processing

**Figure 5:**
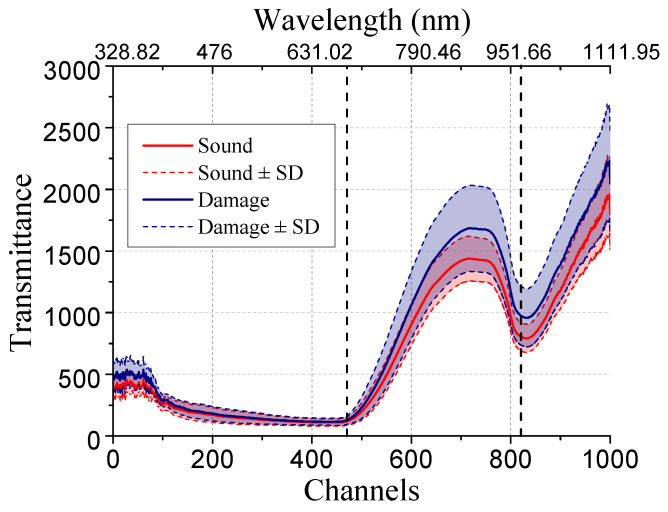
Average transmittance extracted from each channel in image cube.

The raw image in this dataset need to be sub-sampled before use. Figure 5 shows the data structure of hyperspectral transmittance image cube. Feeding image cube with the whole 1002 channels into CNN is unreasonable, since excessive input data points will bring redundant parameters to be trained, which easily leads to overfitting. The unstable average transmittance spectra locating on the first and last few channels in the original data will affect the robustness of the model. Besides, the adjacent channels are similar and hence there exists redundancy caused by high linear relation. Based on the above analysis, we choose the 470 th channel to the 820 th channel, and sub-sample with 5 spectral intervals. We then obtain an image cube of 71 channels with a spectral range from 686.45 nm to 967.77 nm. To reduce computation amount, all the resulting images are further resized to the resolution of 32 × 32.

Unlike RGB images, the pixel value of hyperspectral images ranges from 0 to tens of thousands. In this blueberry dataset, the value of the reflective area is much more higher than that of other areas. However, the amount of information in the reflection area is not large and the extremely high pixel value may affect the robustness of the model. Thus, nonlinear transformation is performed for hyperspectral images. For the $c^{th}$ channel of image cube $y_{i}$, the nonlinear transformed $c^{th}$ channel $y_{i,c}^{\prime }$ is defined as:

(6) $$y_{i,c}^{\prime }=\log_{10}y_{i,c}$$

Then, we zero center every image cube with a specified mean and scale each sample by the specified standard deviation. The mean and standard deviation are evaluated per wavelength channel. The zero-mean normalized cth channel of image cube $y_{i}$ is $y_{i,c}^{\prime \prime }$, presented as follows:

(7) $$y_{i,c}^{\prime \prime }=\frac{y_{i,c}^{\prime }-mean\left(y_{i,c}^{\prime }\right)}{\sqrt{var(y_{i,c}^{\prime })}}$$

Finally, data augmentation is implemented to $y^{\prime \prime }$. Each image is flipped vertically, flipped horizontally, and rotated by 90°/180°/270°. The expanded sample size is six times that of the original training set.

### 3.4. Adjusting the Structure of CNN

Residual Network (ResNet) [30] is used in this classification. In ResNet, hypercubes selected by active learning criteria with resolution of 32 × 32 and 71 channels are fed into the deep neural network. The first convolutional layer aims to mix the original image channels before the data enter the residual blocks. Subsequently, there are 27 residual blocks with different numbers of input and output channels followed by a global average pooling layer and a fully connected layers activated by softmax. With the shortcut connection module in the residual block in ResNet, the output of each layer is not the map of the inputs, but the sum of the inputs and its mappings. The shortcut connection adds the priori information to the latter layers. In the training process, reasonable prior information will promote the model performance.The Rectified Linear Unit (ReLU) function is used as the activation function. The cross-entropy loss function along with the momentum optimizer are utilized to minimize the error. To address the overfitting issues, the batch normalization method is performed before each activation function.

All image processing and statistical analysis were executed in Matlab R2014a (The MathWork, Inc., Natick, MA, USA). The deep learning experiment in this study was implemented using TensorFlow framework (Google Inc., Mountain View, CA, USA). All experiments were performed under a Windows 10 OS on a machine with CPU Intel Core i7-7820HK @ 2.90 GHz, GPU NVIDIA GeForce 1080 with Max-Q Design, and 8 GB of RAM.

### 3.5. Performance Validation

**Figure 6:**
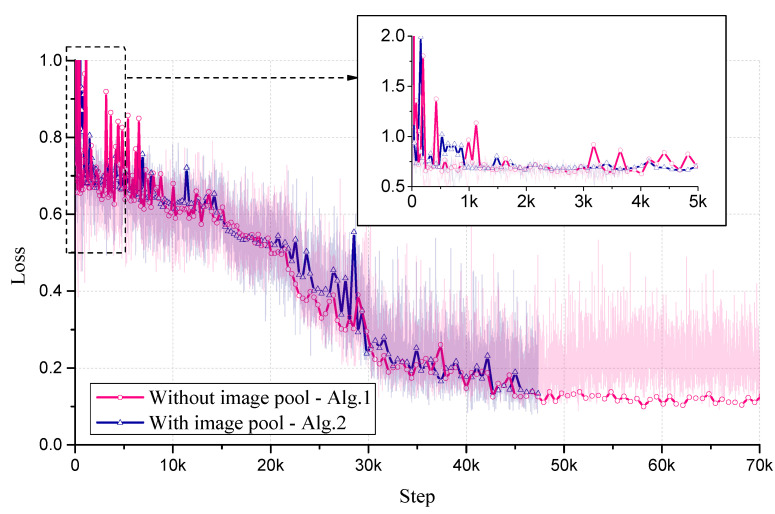
Loss curves of the two algorithms. Each symbol on the curve represents an iteration.

Figure 6 shows loss curves of the two algorithms. In the first few iterations, the model converges slowly and loss value fluctuates severely. As the training process progresses, model converges and the performance tends to be stable.

**Figure 7:**
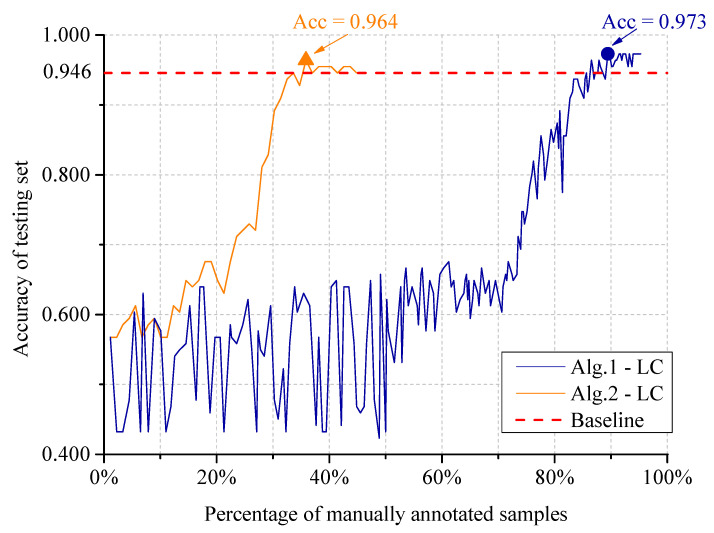
Comparison of Algorithms 1 and 2 using different percentage of training annotated samples, with the active learning algorithm of least confidence (LC). In the baseline model, the whole training set are manually labeled.

Figure 7 presents the classification accuracy of the two algorithms using different percent of annotated samples for training. In the baseline model, all the training set are manually annotated. The active learning algorithms achieve even better performance with less annotated training samples, establishing a cost-effective classification model. In practical application, users can terminate the training process after exceeding the budget for manual annotation or reaching the expected time limit. Meanwhile, they can still obtain a classifier with relatively good performance. In Algorithm 1, manually annotating 85% of the whole training set will achieve the performance of the baseline model. The peak accuracy reaches 0.973 when 89.5% of the whole training set are manually annotated. In Algorithm 2, we introduced image pools to make full use of the manual-annotated samples. The result shows that this modification improves model performance dramatically, manually annotating only 33% of the whole training set can reach the accuracy of baseline model. The peak accuracy reaches 0.964 with 35.9% of the training set are manually annotated.

**Figure 8:**
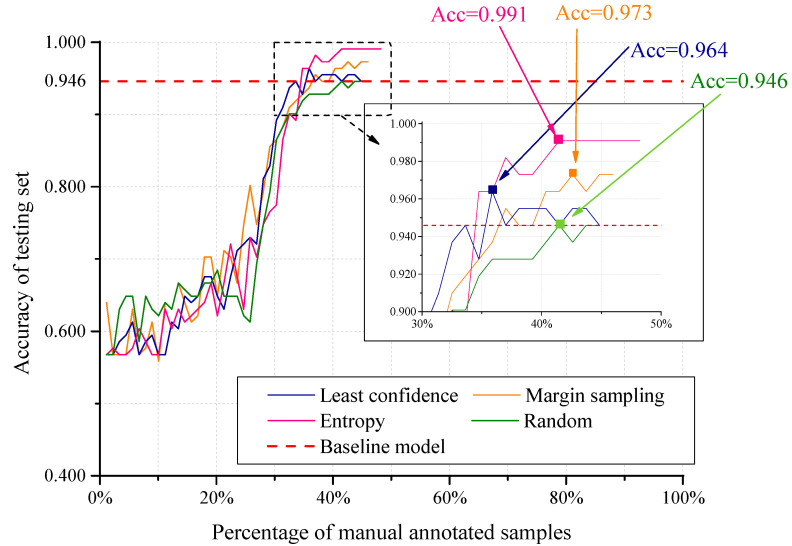
Comparison of least confidence, margin sampling, entropy and random selection.

Figure 8 compares three active learning criteria and random selection based on Algorithm 2. For least confidence, the peak accuracy reaches 0.964 when 35.9% of the whole training set are manually annotated. For margin sampling, the peak accuracy reaches 0.973 when 42.6% of the whole training set are manually annotated. For entropy, the peak accuracy reaches 0.991 when 41.5% of the whole training set are manually annotated. All the three active learning algorithms achieve better performance with fewer manually annotated training samples. In order to learn the contribution of the active learning criteria, we replace active learning module with random selection for manual and pseudo annotation, remaining all the other parameters unchanged. The results show that random selection achieve a lower peak accuracy with more samples manual annotated.

Table 1 presents the comparison of using three active learning criteria based on Algorithms 1 and 2. The introduction of image pool reduces the amount of manual annotated samples effectively, and active learning criterion of entropy can choose training samples with more information comparing with the other criteria.

## 4. Conclusions and Future Work

In this study, we propose a framework using active learning and deep learning in tandem for the multichannel image. Active learning algorithms are introduced as criteria to select informative samples for manual annotation and easy-to-learn samples for pseudo labeling. A total of three active learning algorithms are utilized, i.e., least confidence, margin sampling, and entropy. In the case study on agricultural hyperspectral image classification of blueberries, the proposed framework shows great performance. The combination of Algorithm 2 and entropy achieves accuracy of 0.991 with only manually annotating 41.5% of the whole training set. Furthermore, we introduce an “image pool” to make full advantage of the images generated by data augmentation. The results show that this improvement reduces the amount of manually annotated images used for training by more than a half while guaranteeing the prediction accuracy. In the practical application, the proposed framework can help us establish model with a very low labeling cost, which can be applied to the multichannel image classification in agricultural and biological engineering.

## Figures and Tables

**Table 1:** Comparison of using three active learning criteria based on Algorithms 1 and 2, where LC, MS and EN represent least confidence, margin sampling and entropy, respectively. In baseline model, all the training set are manually labeled.

Method		Algorithm 1			Algorithm 2		
		LC	MS	EN	LC	MS	EN
Baseline	Percentage ^1^	85.7%	86.8%	61.8%	33.6%	36.4%	34.5%
	Accuracy	0.946					
Peak	Percentage ^1^	89.5%	89.2%	73.5%	38.1%	42.6%	41.5%
	Accuracy	0.973	0.973	0.991	0.964	0.973	0.991

^1^ Percentage of manual-annotated samples in training set.
